# Pharmacokinetics behavior of four cannabidiol preparations following single oral administration in dogs

**DOI:** 10.3389/fvets.2024.1389810

**Published:** 2024-04-25

**Authors:** Sasithorn Limsuwan, Natthaporn Phonsatta, Atikorn Panya, Rathapon Asasutjarit, Natthasit Tansakul

**Affiliations:** ^1^Institute of Food Research and Product Development, Kasetsart University, Bangkok, Thailand; ^2^National Center for Genetic Engineering and Biotechnology (BIOTEC), National Science and Technology Development Agency (NSTDA), Pathum Thani, Thailand; ^3^Thammasat University Research Unit in Drug, Health Product Development and Application (DHP-DA), Department of Pharmaceutical Sciences, Faculty of Pharmacy, Thammasat University, Pathum Thani, Thailand; ^4^Department of Pharmacology, Faculty of Veterinary Medicine, Kasetsart University, Bangkok, Thailand

**Keywords:** pharmacokinetics, CBD, cannabidiol, hemp, dog, cannabis

## Abstract

Cannabidiol (CBD) is a natural phytochemical agent and one of the most abundant found in *Cannabis sativa*. It is known to exhibit pharmacological properties on various condition such as relieving-inflammation, pain, epilepsy, and anxiety effect. There has been an increasing trend globally in the use of CBD as a supplement in pets. Consequently, there are various CBD products being marketed that are specifically available for pets. Veterinarians and pet owners are concerned that following ingestion, different CBD formulations may result in a CBD level circulating in the blood that may affect the safe use and efficacy of CBD in pets. Several pharmacokinetics studies in animals have been mainly conducted with an oily form of CBD. To date, there is a lack of data regarding direct comparisons in animals among the CBD plasma kinetic profiles from an oral administration of the various preparation forms. Therefore, the current study evaluated and compared the plasma CBD levels from a single oral administration using four different CBD preparations—liquid (an oil-based form, a nanoemulsion form, or a water-soluble form) or a semi-solid form (as CBD mixed in a treat) in dogs. In total, 32 healthy, crossbreed dogs were randomly assigned into 4 groups and treated according to a 1-period, 4-treatment parallel-design. The three liquid forms were dosed at 5 mg/kg body weight, while the single semi-solid form was given at 50 mg/treat/dog. The results showed that the CBD plasma profile from the administration of a water-soluble form was comparable to that of the oil-based group. The nanoemulsion-based form tended to be rapidly absorbed and reached its peak sooner than the others. However, the CBD in all preparations reached the maximum plasma concentration within 3 h post-dose, with an average range of 92–314 μg/L. There were significant differences among certain parameters between the liquid and semi-solid forms. This was the first study to provide pharmacokinetics data regarding CBD in water soluble, nanoemulsion-based, and semi-solid forms for dogs as companion animals. The current data should facilitate the scrutiny of CBD plasma profiles based on different formulations via an oral route in dogs.

## Introduction

Since discovering the endocannabinoids system and its receptor in the late 1960s, there have been extensive studies to understand the associated mechanisms, functions, and chemical interrelationships (1, 2). Cannabinoids are chemical compounds, mainly produced by *Cannabis sativa L*., that reportedly interact with the endocannabinoids system and exert a biological effect in mammals (1). There are more than 90 compounds in 10 subclasses that have been classified as phytocannabinoids (2). Among these, cannabidiol (CBD), a non phychotropic component of cannabis, has been of interest for its potential use to cure diseases and improve the quality of animal life (3). There have been numerous publications on the *in vitro* and *in vivo* pharmacological effects of CBD in humans and animals, such as anti-inflammatory, analgesic, dermatological and immunomodulation properties (2–5). Specifically, early study of administering CBD in dogs has demonstrated its potential in the alleviation of pain and the clinical symptom of osteoarthritis (6). Recently, it was reported that giving a low dose of CBD in conjunction with an analgesic protocol in horses showed satisfactory pain relief with improved quality of animal life (7). Clinically, the efficacy has been described of CBD to reduce the frequency and severity of seizure in an epileptic dog (8, 9). In addition, CBD has been shown to be useful as adjunctive treatment to relief pruritus in a dog with atopic dermatitis (10).

Despite CBD having substantial therapeutic potential in animals, its pharmacokinetics (PK) profiles in companion animals, especially dogs, have yet to be clearly described (11). CBD is a chemically lipophilic molecule with poor and variable absorption (12). Oral bioavailability of CBD in dogs has been reported to be lower than 20% (13). Notably, it was hypothesized that the first-pass metabolism is one of major concerns regarding the low bioavailability of CBD via oral administration (14). Therefore, there has been much interest to increase the CBD plasma level and to identify alternative routes and different dosage forms of administration.

Several dosage forms of CBD have been studied in animals (11), such as liquid oil-based, capsules, soft chew (15–17), microencapsulated oil beads and transdermal cream (18), intranasal, and as a suppository (19). Nowadays, there are variety of CBD products available for pets, with growing consumption in the global market (11, 20). CBD oil-based preparation is one of most common forms consumed orally by pets and its kinetics behavior has been studied (11). However, the highly lipophilic property of oil-based products affects the CBD level for optimal biological effect, since it has low aqueous solubility and bioavailability. Other CBD options have been developed to improve CBD solubility and delivery into blood circulation and target tissue, such as water-soluble and nanoemulsion forms (5, 21–23). Comparison of the PK profiles of different water-soluble and oil-based preparations has been studied in humans and has confirmed the influence of CBD preparation on its bioavailability (5). Pharmacokinetics describe the time-course concentration of a drug throughout the body and can be utilized as an interpretive and predictive tool of exogenous chemical behavior. The fate of any drug may change based on the site of administration, formulation, and dosage. The PK profiles of different dosage forms in target animals should be studied by taking into consideration the various factors that affect the plasma CBD level.

The scope of the current study was to determine the optimum CBD level using GC-TQ/MS, with the main aim to evaluate the CBD plasma kinetic profiles in mature crossbreed dogs. For this purpose, the study investigated a single-dose CBD administration of four different dosage forms CBD infused in an oil base (OM), a nanoemulsion base (NM), a water-soluble base (WM), and a semi-solid form as a treat (CM). The current investigation should provide insights that are relevant to prudent use and practice on CBD delivery and to efficacy strategies via oral delivery in dogs.

## Materials and methods

### Chemical and CBD preparation

The CBD standard was purchased from Cerliliant^®^ (product code: 13956–29-1). Certified CBD powder was obtained from Salus Bioceutical (Thailand) Co., Ltd. with purity greater than 99%, as reported by a certified test laboratory third party. HPLC and LC/MS grades of acetonitrile and methanol were purchased from Labscan Co. Ltd. (Bangkok, Thailand).

CBD in oil-based was prepared by dissolving CBD powder in natural virgin coconut oil (100% cold-pressed). In brief, 1.5 g of CBD isolated powder were weighed into a volumetric flask and dissolved in 30 mL of oil and dispersed using a magnetic stirrer on a warm plate at approximately 45°C for 30 min.

The nanoemulsion formulation was not the main objective in this study. Therefore, a test of the potential of a nanoemulsion for CBD delivery was performed following a method developed in-house for oral herbal oil formulations. In short, an oil-in-water nanoemulsion was prepared using a high-pressure homogenization technique (15,000 psi, 5 cycles), comprising oil droplets with diameters in the range 150–200 nm. The nanoemulsion was achieved by mixing an aqueous phase (comprising purified water, propylene glycol, sodium EDTA, paraben concentrate, Tween 80) and an oil phase (comprising CBD, short and medium chain triglycerides, alpha-tocopherol, and Span 80).

The water-based CBD comprised 20% CBD water-soluble powder in a modified blended starch of corn and tapioca as an emulsifier. Briefly, 7.5 g of CBD water-soluble powder were added to a 50 mL volumetric flask. Then, 30 mL of purified water was added and mixed using a magnetic stirrer for 15 min to achieve a final concentration of 50 mg/mL. Notably, prior to administering liquid forms to each animal, the CBD concentration of each preparations was re-assayed using HPLC and the volume was corrected where necessary for a 5 mg/kg dosage. In short, the HPLC- DAD (Thermo Scientific™ Vanquish™ Core HPLC systems) in-house validation method for quantification of CBD showed linearity over the range 0.01–0.4 mg/L, with a coefficient of determination ≥0.999 and a LLOQ of 0.01 mg/L. The percentage values for precision and accuracy were within 3.60–4.18% and 95.6–102.4%, respectively.

The CBD in treat form was prepared by mixing small pieces of the ingredients (corn, rice bran, coconut oil and water). Then, the mixed result was individually loaded to provide CBD oil at 50 mg/treat. All treat samples were placed in an oven at 100°C for 30 min. To prove the CBD level in the treat, 10 treat samples of the same batch were sampled, with the results showing that the CBD level in all samples was at the expected concentration with a standard deviation of less than 1.8%. All CBD preparations were kept in well-sealed containers and placed in a refrigerator (4°C) before being used for animal ingestion within 7 days.

### Animals and ethical considerations

The study was performed in accordance with the permit from the Committee for the Approval of Animal Care and Use for Scientific Research of the Faculty of Veterinary Medicine, Kasetsart University, Bangkok, Thailand (approval number ACKU 62-VET-058).

In total, 32 healthy, crossbreed intact dogs with individually numbered identification (aged 1–5 years, weight 11–23 kg) were equally randomized into 4 parallel design treatment groups (4 males and 4 females in each group). The animals had not been treated with any medication during the previous 4 weeks and were acclimatized for at least 14 days prior to treatment. The animals were housed in separate kennels, with the housing conditions and animals being managed in accordance with the standard of operation of the University. Physical examination, clinical observation, hematology, and blood chemistry were carried out during acclimatization. All animals were fasted overnight before dosing. Any indications of relevant clinical signs or adverse events were observed twice daily for 3 days pre- and post-treatment.

### Dosing design

A single oral dose of CBD in liquid forms: for an oil base (OM), a nanoemulsion base (NM), a water-soluble base (WM), was given to each fasted animal based on the animal’s actual body weight (BW) with the target being 5 mg/kg BW in individually adjusted dosage volumes. In the semi-solid form (CM), each serving contained 50 mg of CBD per dog that was given by hand directly into the month of the fasted animal for self-ingestion with a tray underneath that collected any spilled pieces of test item, which were then reinserted into the animal’s mouth cavity and the animal was carefully observed to ensure all the treat had been swallowed.

### Specimens and collection

Blood samples were collected and stored in a tube containing lithium heparin via cephalic or saphenous venipuncture with a no. 22” IV-catheter at the following time points: −1 day, 0, 30 min, and then 1, 2, 3, 6, 10, 24, and 30 h after a single oral ingestion. Following collection, the samples were immediately placed on ice and protected from direct light until centrifugation. After centrifugation at 3,000 × *g* for 10 min at a controlled temperature of 4°C, plasma was collected in laboratory-coded, labeled aliquots. Then, the aliquots were transported in an ice-pack box to be frozen at −80°C in a dark cover box pending analysis within 65 days.

### Quantitative measurement of plasma-containing CBD

The groups of eight animals per treatment were studied for their plasma concentration-time profiles of CBD using an in-house, validated gas chromatography method modified from previously described (24). In brief, 100 μL of plasma sample were extracted with 400 μL of methanol and then triple-vortexed at 2,200 rpm for 10 min. Samples were centrifuged at 10,000 × *g* for 10 min at 4°C. A portion of the 100 μL of supernatant was transferred into a 2 mL GC glass vial. An internal standard using myristic acid-D27 in hexane (1 μmol/mL) was added for 20 μL. The mixture was dried at 60°C for 2 h, then added with 50 μL dichloromethane, and dried again for 30 min to remove the residual water. For the trimethylsilylation reaction, a modified method was performed following an assay described (24, 25). In brief, 50 μL of N-methyl-N-(trimethylsilyl) trifluoroacetamide (MSTFA), containing 1% of trimethylchlorosilane, were added into each mixture and incubated at 37°C for 30 min. The derived samples were cooled at room temperature and transferred into glass vials with micro-inserts and capped immediately for analysis. Each sample was analyzed using gas chromatography triple quadrupole tandem mass spectrometry (GC-TQ/MS; GC 7890B/MSD 7000D; Agilent Technologies; United States) coupled to a PAL3 auto sampler system (CTC Analytics AG; Switzerland).

An injection volume of 2 μL of the derived samples was analyzed using the GC-TQ/MS in split mode with an injector temperature of 250°C, a split ratio of 10:1, with a DB-5MS UI column (30 m, 0.25 mm i.d.; Agilent Technologies; United States). Helium was used as the carrier gas with a constant flow rate of 1.0 mL/min. The GC oven was programmed with an initial oven temperature of 60°C, then ramped from 60°C to 325°C at the rate of 10°C/min, and held for 10 min. The transfer line, ion source, and quadrupole were set at 325°C, 240°C, and 180°C, respectively. The mass spectrometer was operated in dynamic multiple reaction monitoring (dMRM) mode and detected for transition at m/z 389.9 > 301.2 m/z for CBD and 312.0 > 119.9 > 73 m/z for myristic acid-D27. To achieve acceptable precision and accuracy for CBD quantification, the derivatized samples were limited to 30 samples (injections) a day with a proper moisture removal procedure. All samples were stored at <10°C using a PAL3 Peltier stack and tray to ensure the stability of the targeted compounds. In addition, the mass detector tuning and calibration curve were performed daily before commencing the new sequence operation. Data were acquired using the MassHunter software (version 10.0; Agilent Technologies; United States) based on three replicates to calculate the mean and the standard error. The calibration curves were determined using CBD at different concentrations in the range 1–800 ng/mL in plasma with myristic-d27 acid as an internal standard (co-efficient of determination = 0.9999). The quantitative analyses were performed using the Agilent MassHunter software (version 10.0; Agilent Technologies; United States) and exported into the Excel software (Microsoft Corp.; United States) for further data processing.

### Pharmacokinetic evaluation

The pharmacokinetic parameters in this study were evaluated following a typical model-independent approach using non-compartmental analysis (NCA). NCA was performed using the R software [version 4.3.2; R Core Team, (2022-10-31)], focusing on the key parameters of maximum plasma concentration (C_max_), time to maximum concentration (T_max_), area under the curve to the last quantifiable time-point (AUC_0-t_), area under the curve extrapolated to infinity (AUC_0-inf_), terminal phase elimination rate constant (Ke), apparent volume of distribution during the terminal phase (Vz/F), apparent clearance (CL/F) after non-intravenous administration (assuming that the ratio of clearance to bioavailability is constant without IV comparison), mean residence time extrapolated to infinity (MRT_inf_), and elimination half-life (T_1/2_). The dose normalized C_max_ and AUC parameters were calculated to facilitate the assessment of dose proportionality. The relative bioavailability values were calculated following dose-normalization using AUC_(another form)_/AUC_(OM form)_ × 100.

### Statistical analysis

Microsoft Excel and GraphPad Prism version 9.5.1 (733) for Windows (GraphPad Software; United States) were used to calculate descriptive and inferential statistical analyses (where applicable), including all outcome calculation data of the middle of a dataset (median values), measure of central tendency (average; mean), variability (standard deviation, standard error mean) and figures. Normality distribution was determined using the Shapiro–Wilk test, while differences between groups for the AUC_0-t_, C_max_, T_max_, and Ke parameters were analyzed using a Brown-Forsythe ANOVA following a *post hoc* Dunnett’s T3 multiple comparisons test. Non-normality distributions for the T_1/2_, AUC_0-inf_, Vz/F, CL/F and MRT_inf_ parameters were tested using the Kruskal-Wallis with a *post hoc* Dunn-Bonferroni test to achieve pairwise multiple comparison data. A value of *p* ≤ 0.05 was defined as significant.

## Results

This study was conducted to investigate the pharmacokinetics of CBD in crossbreed intact dogs following a single dose. An in-house validation of the GC-TQ/MS method for fortified dog plasma was achieved with an instrument detection limit at 0.05 μg/L and a LLOQ of 1 μg/L blood plasma, with satisfactory intra-day precision based on coefficient of variation and accuracy results in the ranges 5.8–10.8% and 85.2–110.3%, respectively. Inter-day precision and accuracy were in the ranges 9.8–10.1% and 92.6–102.45%, respectively.

**Figure 1:**
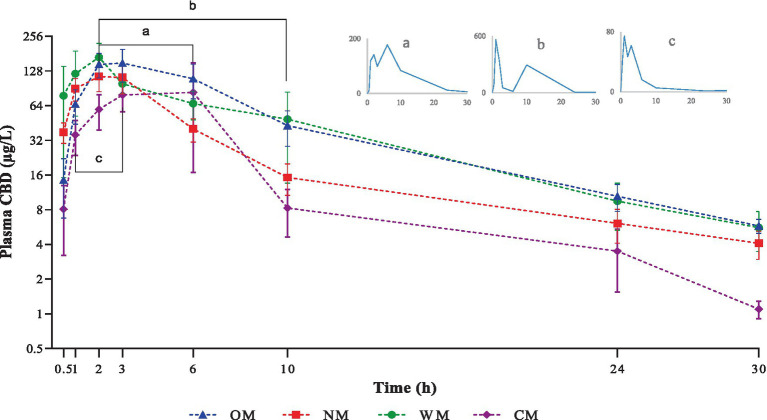
Graphical representation of semi-logarithmic scale of CBD plasma levels (mean ± SEM) after single oral dose administration of one of four different dosage forms: CBD infused in an oil base (OM), a nanoemulsion base (NM), a water-soluble base (WM), or a semi-solid form (CM). Line bar and sub-figures with letter a, b and c indicate time-points where secondary-peaks were observed in one dog of the OM, WM and CM groups, respectively.

**Figure 2:**
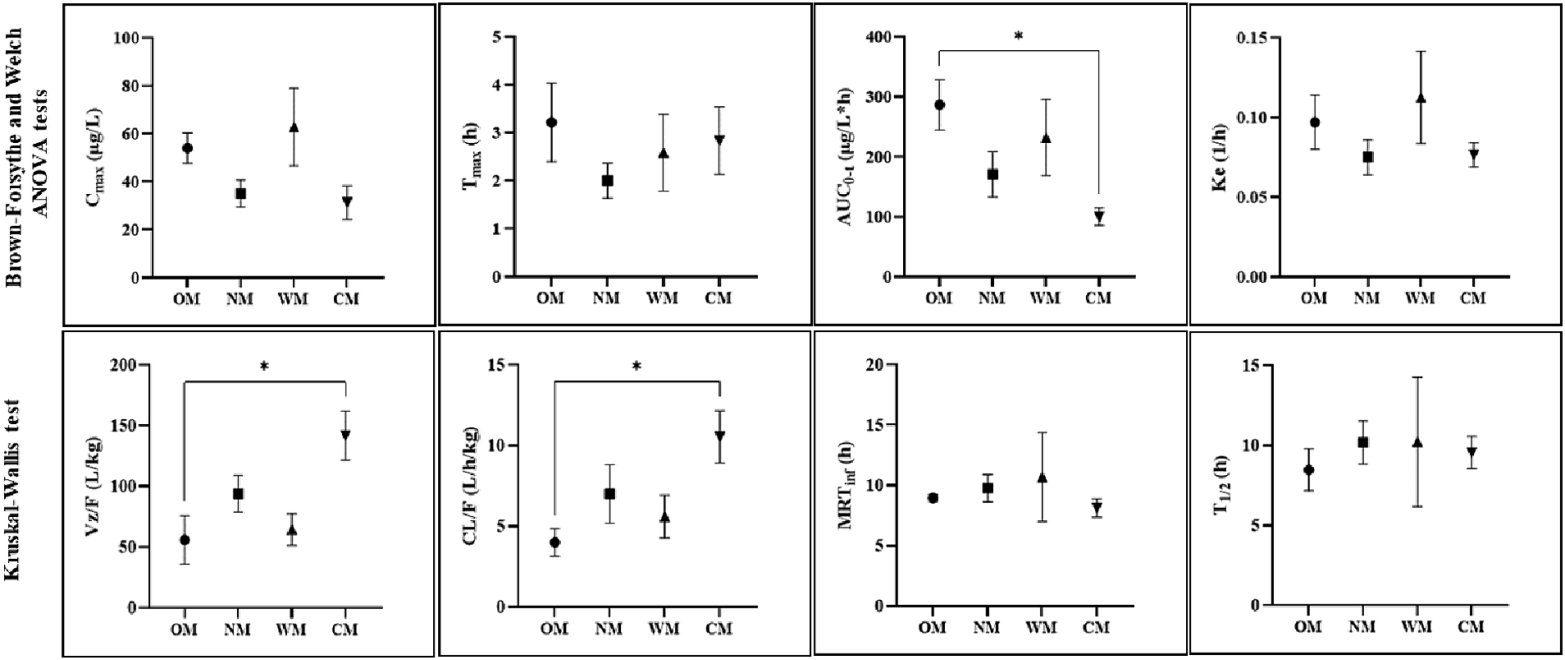
Representative PK parameters (mean ± SEM) for four different forms: CBD in an oil base (OM), a nanoemulsion base (NM), a water-soluble base (WM), a semi-solid form (CM), with significance indicated by ^*^p < 0.05 (dose-normalized: mg/kg, C_max_ and AUC parameters).

The treatment involved the dogs receiving one of either a single dose of liquid form CBD infused in an oil base (OM), a nanoemulsion base (NM), or a water-soluble base (WM), or of semi-solid form as treat (CM). All animals completed this experiment with no adverse clinical events occurring during the study. At the studied dose, there were no signs of serious gastrointestinal or nervous disorders in the dogs during and post dose. A single intake of each serving contained 50 mg CBD for each dog in the CM group. The data set of dose-normalized C_max_ and AUC parameters were compared to other groups. Notably, only one dog in the CM group appeared to produce more saliva than usual when chewing, but recovered soon after ingestion. The root cause of this was not identified. The PK parameters, using non-compartmental analysis, of CBD in the 4 preparations following a single oral administration to overnight fasted dogs are summarized in Table 1. The plasma concentrations of CBD (mean ± SEM) for each time point of all groups were calculated and are presented as a semi-log graph in Figure 1. Certain PK parameters were statistically significant, as shown in Figure 2. Indeed, following the pharmacokinetic estimation, a dog in the OM group and two dogs in each of the remaining groups were excluded in the subsequent descriptive summary due to insufficient data points in the elimination phase. In addition, the excluded data resulted in inaccurate estimation of the Ke, T_1/2_, Vz/F, and extrapolation of AUC_0-inf_. It could also affect calculations of MRT and CL/F, as they are calculated using AUC_0-inf_.

**Table 1:** PK parameters (mean ± SEM) of CBD following single oral dose administration of one of four different dosage forms.

Pharmacokinetic parameter	OM (*n* = 7)			NM (*n* = 6)			WM (*n* = 6)			CM (*n* = 6)			*p*-value
AUC_0-t_ (μg/L*h)	1432.06	±	208.38	853.29	±	188.83	1158.98	±	317.83	296.05	±	41.22	0.0431^a^
AUC_0-inf_ (μg/L*h)	1494.14	±	209.87	935.19	±	200.42	1308.98	±	378.85	313.84	±	41.92	0.0381 ^a^
C_max_ (μg/L)	270.10	±	31.88	175.35	±	28.19	314.30	±	81.09	92.29	±	21.45	0.1329^a^
T_max_ (h)	3.21	±	0.82	2.00	±	0.37	2.58	±	0.80	2.83	±	0.70	0.6584
T_1/2_ (h)	8.47	±	1.31	10.19	±	1.35	10.23	±	4.05	9.56	±	1.01	0.4796
Ke (1/h)	0.10	±	0.02	0.08	±	0.01	0.11	±	0.03	0.08	±	0.01	0.4467
Vz/F (L/kg)	55.94	±	19.80	93.93	±	15.01	64.48	±	12.88	141.75	±	20.30	0.0199
CL/F (L/h/kg)	4.00	±	0.85	7.00	±	1.80	5.59	±	1.31	10.54	±	1.61	0.0381
MRT_inf_ (h)	8.96	±	0.27	9.77	±	1.11	10.69	±	3.66	8.14	±	0.75	0.5032

a A value of *p* ≤ 0.05 was defined as significant and indicated the data with dose-normalization where applicable.

CBD infused in an oil base (OM), a nanoemulsion base (NM), a water-soluble base (WM), or a semi-solid form (CM) in the OM, NM, WM and CM groups, respectively, where *p* values are based on *post hoc* multiple comparisons using Brown-Forsythe ANOVA and otherwise (underlined) using non-parametric Kruskal-Wallis test.

Following dose-normalization, the results showed no significant difference between the C_max_ of the CBD in the plasma after administration of all groups. However, the highest C_max_ of the CBD in the plasma (314.30 ± 81.09 μg/L) was obtained from administration of the WM group, while the lowest C_max_ of the CBD in the plasma was in the CM group (92.29 ± 21.45 μg/L).

The values for the mean AUC_0-t_ and AUC_0-inf_ of the OM formulation were 1432.06 ± 208.38 and 1494.14 ± 209.87 (μg/L*h), which displayed the highest extent of CBD exposure compared to the other treatments. The CM group provided the lowest extent of CBD in the plasma of around 296.05 ± 41.22 and 313.84 ± 41.92 (μg/L*h) for the mean of last quantifiable time-point and the curve to infinite time, respectively. The relative bioavailability levels after dose-normalization of the other formulations comparing to the OM formulation were 80.9, 59.5, and 34.8% for the WM, NM and CM groups, respectively.

Among the preparations, there was rapid absorption in the NM group, with peak plasma concentrations occurring within 2 h after ingestion. The CBD plasma concentrations of the liquid forms reached a peak over 100 μg/L within 6 h in all dogs. In contrast, only three of the eight dogs in the CM group achieved a C_max_ over 100 μg/L. The exposure using the semi-solid dosage form (CM) represented by the AUC was much lower than for the other dosage forms.

The T_1/2_ of plasma CBD in the OM group was 8.47 ± 1.31 h, which was shorter than for the others but not significantly different. At 30 h post-dose, CBD was detectable in all dogs in the OM group but it was not detected in 1 out of the 8 dogs in each of the NM, WM, and CM groups. All the MRT_inf_ had a similar level range (8.14–10.69 h). There were significant differences between the liquid and semi-solid forms for certain parameters (AUC_0-t_, Vz/F, and CL/F). As a result, the kinetic profiles of the CBD in the liquid forms were relatively similar, particular for the OM and WM groups. As such, the results demonstrated that the main PK parameters of the CBD within liquid forms were not as straightforward as anticipated. The impact of the dosage form is covered in the discussion below.

## Discussion

Utilizing cannabis-related products to achieve favorable health impacts is rapidly increasing following its legalization in some parts of the world. In June 2022, Thailand removed marijuana from its narcotics list and became the first Asian nation to approve cannabis for medicinal and industrial use (26). Cannabis use is of interest not only in human medicine, but also in veterinary medicine, where extensive research is warranted to better understand the behavior and impact of the drug after administration in animals, since interspecies differences are a main factor influencing PK variation (27). Dogs have been the main companion animal species studied; however, the published PK studies have mainly been on oil-based CBD (11, 15, 28).

The current study was designed to explore the PK patterns of CBD in the plasma from different preparation forms—liquid (the OM, NM, and WM groups) and semi-solid (the CM group)—following a single oral administration in overnight fasted crossbreed dogs. It is known that CBD is a lipophilic compound with limited absorption into circulating blood (12). Several reports on the PK of CBD in dogs have studied CBD in oil-based formulations, microencapsulated-beads, chewable soft capsules, and soft gel capsules (11, 15–18). However, comparison data of oral CBD profiles in companion animals with different formulations are scarce. To the best knowledge of the authors, this is the first report on CBD in nanoemulsion and water-soluble forms in dogs. In addition, according to the limited available information on CBD in semi-solid form, this study has presented plasma CBD behavior following snack-as-treat ingestion in dogs.

Variation in the PK pattern of plasma CBD arises from an extensively first-pass metabolism and its low aqueous solubility that leads to poor bioavailability and a poor biological effect (12, 14). Commonly, inconsistent and variable systemic drug exposure are affected by multiple factors, including route of administration, dosage form, dose range, and health and feeding status. It has been noted that differences in the study design, including animal signalment (breed, sex, age) and status, sampling time point, and determination method, may affect PK outcomes; therefore, it is inappropriate to directly compare those estimated PK parameters between various experiments (29).

The oral bioavailability of CBD in dogs has been estimated to be in range 13–19% (13). Improving CBD delivery into the blood stream and its efficacy via an oral route is challenging to achieve a therapeutic response. Specifically, numerous studies have been conducted with various developed CBD preparations to increase the oral bioavailability and PK evaluation in dogs (11, 15–18).

The current results indicated there were no significant differences between all PK profiles across the CBD delivered in liquid form. However, comparison of the CBD in liquid forms against the semi-solid form identified differences in the Vz/F, CL/F, AUC_0-t_, and AUC_0-inf_ parameters. The current findings showed that the CBD behavior profiles in the WM group were comparable to those in the OM group. Although the highest C_max_ was in the WM group, it was a noticeably high variation, including for the MRT_inf_, T_1/2,_ and AUC parameters. This might indicate that the estimated rate of absorption in water-soluble dosage form had larger bioavailability variation than for the oil-based form. Coincidentally, another study reported that a water-based formulation of CBD, which had a similar composition to the WM prepared in the current study also reported statistically comparable PK parameters in human plasma compared to that of the CBD in human plasma after oral administration of the oil-based formulation of the CBD (5).

There has been a wide range reported of the CBD maximum concentration following oral administration of oil-based CBD formulations (11). Compared to another experiment, in which there was oil-based administration at the same dose in fasted dogs, the C_max_ value in the OM group in the current report was about twice that of the earlier report (30). In contrast, another experiment that involved drug administration to fed dogs with an equal adjusted dose of CBD-infused oil had a C_max_ that was around twice that of the current study (18).

Several factors influence the bioavailability and disposition of CBD, resulting in relatively high intra- and inter-individual variability in the PK profiles. Co-consumption of CBD with food, particularly in a fat meal, may alter the rate and extent of absorption. It has been reported that in humans, CBD plasma levels increased when concomitantly administered with food or in a fed state (31, 32). Likewise, positive food effects have been associated with increased maximum systemic exposure without affecting the AUC_0-t_ in dogs; however, higher C_max_ and AUC levels were observed in one out of three fasted dogs (30). Contrary to these results, Vaughn et al. (20) argued that overnight fasting with dogs might enhance the systemic absorption of CBD. In rabbits, it has been reported that feeding decreased systemic CBD absorption (33). In fact, fasted-fed variability is affected by various factors, such as the physiological condition, demographic and genetic factors, chemical, and formulation-related factors (34).

A single dose administration in the current study presented fluctuations of CBD plasma concentrations both within and between groups. Recently, it has been suggested that giving CBD twice daily may reduce the variation in plasma concentration (28). In addition, the maximum CBD plasma concentration has been reported to increase in a dose-dependent manner but some studies seemed not to be linear (16, 28–30).

Notably, the current findings corroborated the phenomenon of the so called ‘secondary peak’ as it was found in one dog in every group except the NM group. At first, this was considered as a possible error in the sample preparation or related to the laboratory process; however, it was confirmed following double checking and determining with different instruments of detection. The explanation for this phenomenon is not yet clearly understood. The double peak of CBD found in the plasma has been suggested to have been caused by a combination mechanism, such as enterohepatic recycling and intestinal lymphatic absorption (35). In addition, the CBD secondary peak has been reported in dogs given a medium (5 mg/kg) or high (10 mg/kg) dose rather than a low (2 mg/kg) dose, with coprophagia being one possible explanation (29).

However, the absence of a secondary peak of CBD in the dog plasma after orally taking NM form may have been due to the small size of the oil droplets in the nanoemulsion. Consequently, they had a larger surface area and so were efficiently exposed to the intestinal lipase at its binding sites (36). Therefore, the CBD in the oil droplets of the nanoemulsion was absorbed after the oil was digested and rapidly transformed into the primary derivatives (CBD-7-COOH and OH-7-CBD) through a first-pass metabolism process that resulted in a rapid decrease in the CBD concentration in the plasma (37). Finally, the content of the plasma CBD in the NM form could not be detected as a second peak, as occurred for CBD in the other dosage forms.

In all four dosage forms, the CBD was rapidly absorbed with mean maximum plasma concentrations occurring in the range 2–3.2 h post-dose. This was in agreement with other reports, where the times to reach the maximum plasma concentration were within 1–4 h, with no effect of dose amount or duration of exposure (6, 15, 16, 29).

The current results showed that the extent and rate of CBD systemic exposure in the OM group was highly absorbed. The lowest extent of absorption for the CM formulation compared to the other formulations was confirmed by the relative bioavailability value. The low plasma levels of the CM group could have been due to the low oral bioavailability of the semi-solid formulation, considering that the AUC was significantly lower than for the oil-based formulation (OM group), with the value for Vz/F and CL/F being significant higher than for the OM group. CBD degradation from the treats snack in the CM group following the heat process in preparation was ruled out because the CBD concentration was re-checked prior to being given to the dogs. Unlike the semi-solid form preparation in the current experiment, another study found that CBD in a soft chew format had high absorption with a delayed time to reach its peak, confirming that differently formulated preparations affect the PK outcome (15). As such, liquid and solid forms may alter the rate of absorption and total bioavailability.

Notably, CBD in the NM group followed by the WM group peaked sooner than the CBD in the OM group, which may support the rapid onset of an effect. The effect of nanoemulsion based on rapid oral absorption of the CBD found in the current study was consistent with the results reported by Yen et al. (38), who found that an andrographolide-loaded nanoemulsion was rapidly absorbed via the gastrointestinal tract because the surfactant molecules in the formulation (Tween 80 and Span 80) could suppress the function of P-gp, which inhibited drug secretion by the P-gp-mediated efflux process in the intestinal tissue. Therefore, the shorter time to reach the CBD peak from oral administration of the NM group may have been due to the effect of these particular surfactants.

In fact, oral drug absorption depends on the conditions in the gastro-intestinal tract. Consequently, it was possible that fasting the animals overnight in the current study might have shortened the time to peak concentration for the NM and WM forms. The interaction of PK properties and physiological features in the empty gastro-intestinal tract, including enteric epithelium and influx-efflux transporters, may have hindered this phenomenon; however, the mechanism has not yet been well elucidated.

Currently, there is a lack of research into using a nanoparticle-based approach with different techniques and routes of application to enhance CBD uptake (12). Development and commercial scale production of cannabinoid-loaded nanoemulsions have been highlighted to improve the absorption rate and efficacy for therapeutic purposes (39). The current findings showed that CBD in a nanoemulsion-based formulation tended to achieve rapid absorption, avoiding any fluctuations in kinetic behavior. A similar finding was recently reported, whereby a nanoemulsifying-CBD formulation had a shorter time to reach C_max_ compared to CBD in an oil-based form (35). In addition, it has been mentioned that the nanoemulsion formulated may have improved the rate and variability of absorption (40). However, notably, different CBD nanocarriers delivered different C_max_ and T_max_, outcomes at a particular site of action (21).

Little information is available on CBD volume distribution in dogs. The larger values of the apparent volume of distribution in the liquid forms in the current study seem to suggest the CBD was more likely retained in the body than circulated in the blood. The current results had a Vz/*F* value nearly triple that of an oil-based treatment with the same dose in another study (28). In humans, it is evident that CBD is rapidly distributed throughout the tissues resulting in very high volume of distribution (41). Notably, the nanoemulsion-based treatment had a significantly higher volume of distribution compared to the semi-solid form. The modifying mode of delivery in the nanocarrier formulation may have enhanced the dispersion of CBD throughout the body. Clinically, CBD could be administered in multiple doses over several days up to a month for a therapeutic effect (18). Thus, the tissue distribution ratio should be considered of the CBD dispersed among physiological tissue and accumulated in parts of the body (20). There should be further study of the biodistribution of different CBD preparations within therapeutic sites of interest in the target animal.

Despite scarce scientific information, the use of CBD in dogs has been of broad interest to owners, based on anecdotal evidence of its therapeutic benefits. Notably the current study was conducted with a non-Beagle breed which may not be directly comparable to reported studies involving a Beagle breed. However, based on visual assessment, the crossbreed dog PK parameters of CBD did not show any significant differences from those in the study conducted with Beagle dogs (11, 28). The crossbreed dog population is estimated at 31–53% in the United States, Germany, and the UK (42). In Thailand, based on domestic survey data of pet owners, crossbreed dogs constitute approximately 29%. A limitation of the current study was the small number of crossbreed dogs with only 8 per group. An another limitation is that less frequently in blood sample collection after 10 h post-dose which may cause insufficient data points in the elimination phase. Since there is a wide range of crossbreed dogs, with undoubtedly differences in response to drug behavior, it is unclear whether the plasma concentrations in the current study can be considered representative of the general population of crossbreed or different-sized dogs. The differences in the CBD absorption rate and its metabolic action across dogs has resulted in high variability in plasma concentrations (20). The interpretation and practical use of the available pharmacokinetic data from the current single-dose study should be further investigated. Nonetheless, the novelty of this study is the generation of data relevant to different CBD forms and its behavior in dogs.

## Data availability statement

The original contributions presented in the study are included in the article/supplementary material, further inquiries can be directed to the corresponding author.

## Ethics statement

The animal studies were approved by The Committee for the Approval of Animal Care and Use for Scientific Research of the Faculty of Veterinary Medicine, Kasetsart University, Bangkok, Thailand (approval number ACKU 62-VET-058). The studies were conducted in accordance with the local legislation and institutional requirements. Written informed consent was obtained from the owners for the participation of their animals in this study.

## Author contributions

SL: Writing – review & editing, Validation, Project administration, Formal analysis, Data curation. NP: Writing – review & editing, Formal analysis. AP: Writing – review & editing, Validation, Formal analysis. RA: Writing – review & editing, Validation, Methodology, Formal analysis, Data curation. NT: Writing – review & editing, Writing – original draft, Visualization, Validation, Supervision, Software, Resources, Project administration, Methodology, Investigation, Funding acquisition, Formal analysis, Data curation, Conceptualization.
